# Energy Transfer Assisted Fast X‐ray Detection in Direct/Indirect Hybrid Perovskite Wafer

**DOI:** 10.1002/advs.202103735

**Published:** 2022-03-23

**Authors:** Lulu Liu, Weijun Li, Xiaopeng Feng, Chunjie Guo, Huimao Zhang, Haotong Wei, Bai Yang

**Affiliations:** ^1^ State Key Laboratory of Supramolecular Structure and Materials College of Chemistry Jilin University Changchun 130012 P. R. China; ^2^ Department of Radiology The First Hospital of Jilin University Changchun 130061 P. R. China; ^3^ Optical Functional Theranostics Joint Laboratory of Medicine and Chemistry The First Hospital of Jilin University Changchun 130012 P. R. China

**Keywords:** energy transfer process, fast X‐ray detection, hybrid X‐ray detector, lowest detectable dose rate, sensitivity

## Abstract

Metal halide perovskite scintillators encounter unprecedented opportunities in indirect ionizing radiation detection due to their high quantum yields. However, the long scintillation lifetime of microseconds upon irradiation, known as the afterglow phenomenon, obviously limits their fast development. Here, a new type of hybrid X‐ray detector wafer combining direct methylamine lead iodide (MAPbI_3_) semiconductor and indirect zero‐dimensional cesium copper iodide (Cs_3_Cu_2_I_5_) scintillator through low‐cost fast tableting processes is reported. Due to the fast energy transfer from Cs_3_Cu_2_I_5_ to MAPbI_3_, the device response time to X‐rays is dramatically reduced by nearly 30 times to 36.6 ns, which enables fast X‐ray detection capability by a large area detector arrays within 1 s. Moreover, Cs_3_Cu_2_I_5_ exists at the grain boundaries of MAPbI_3_ crystals, and blocks the paths of mobile ions of perovskite, leading to the lowest detectable dose rate of hybrid X‐ray detector is thus reduced by 1.5 times compared with control MAPbI_3_ direct‐type semiconductor, and 10 times compared with the Cs_3_Cu_2_I_5_ indirect‐type scintillator. The direct/indirect hybrid wafer also exhibits improved operation stability at ambient conditions without any encapsulation. This new kind of hybrid X‐ray detectors provides strong competitiveness by combining the advantages of both direct perovskite semiconductors and indirect perovskite scintillators for next‐generation products.

## Graphical abstract

Direct‐indirect hybrid perovskite X‐ray detector is prepared through a fast‐tableting process. Due to the fast energy transfer from Cs_3_Cu_2_I_5_ to MAPbI_3_, the response time of the hybrid device is dramatically reduced from 1 µs to 36.6 ns, which promotes the development of X‐ray fast dynamic imaging. This new kind of hybrid X‐ray perovskite detector provides an opportunity for existing scintillators with serious afterglow phenomenon.

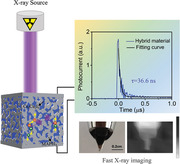

## 1 Introduction

Sensitive X‐ray detectors are widely deployed in medical examination, radiotherapy, product quality inspection, security checks, aerospace navigation, and other fields due to the unique penetration capability of X‐rays.^[^ ^1^, ^2^, ^3^ ^]^ X‐ray detectors basically convert X‐ray photons into electrical signals, and are collected by the readout integrated circuit (ROIC).^[^ ^4^, ^5^ ^]^ According to the detection mechanism, X‐ray detectors are divided into indirect‐type scintillators and direct‐type semiconductors.^[^ ^6^, ^7^, ^8^ ^]^ Direct‐type semiconductor attenuates the X‐rays, generates electron‐hole pairs, and then outputs the charge signals under a large electric field. Advanced direct‐type detector materials include Si, *α*‐Se, Ge, Cd_1‐x_Zn_x_Te (x is <20%, denoted as CZT), and so on.^[^ ^9^, ^10^ ^]^ However, Si and *α*‐Se are limited by their low atomic number and low attenuation coefficients, and the complex integration process of CZT involves technical difficulty and increased cost. In recent years, halide perovskite semiconductors have been discovered as excellent candidates for X‐ray detectors in enhancing the sensitivity in direct detection mode for low dosage imaging due to their strong stopping power, large mobility‐lifetime product (µ*τ*), low defect density, tunable bandgap, easy synthesis method, etc., which are even superior to a commercial *α*‐Se semiconductor.^[^ ^11^, ^12^, ^13^, ^14^, ^15^, ^16^ ^]^ Next milestone of a direct‐type perovskite X‐ray detector can be expected toward large‐area flat‐panel integration for fast medical CT imaging. However, one big obstacle faced for perovskite semiconductors is the serious ion migration effect of perovskite ions, especially under a large electric field to extract the charges induced by X‐rays, thus impairing the device signal‐to‐noise ratio (SNR) and accelerating the degradation of perovskite‐based electronic devices.^[^ ^17^, ^18^ ^]^

Indirect‐type X‐ray detectors based on scintillation crystals first convert X‐rays into UV–vis photons, and followed by photodetector detection through image sensors, such as complementary metal‐oxide semiconductor (CMOS), charge‐coupled devices (CCDs), thin‐film phototransistor (TFT) arrays, and so on,^[^ ^19^, ^20^ ^]^ which avoids applying large bias and ions migration effect. The development of indirect‐type X‐ray detection is relatively mature, and now dominating the markets of CT products. However, there are only limited scintillators available due to the stringent requirements of ionizing radiation detection. For example, some classic scintillators such as NaI (Tl), CsI (Tl), YAlO_3_ (Ce), and Bi_4_Ge_3_O_12_ crystals are grown by the Czochralski method at temperatures above 1700 °C, and the fabrication conditions are harsh and expensive.^[^ ^21^, ^22^ ^]^ In addition, UV–vis light will be scattered in the scintillation crystal, which seriously limits the collected quantum efficiency and spatial resolution due to the cross‐talk problem at neighbor pixels, and increases the difficulty and cost when coupled with CMOS or CCDs image sensors.^[^ ^23^ ^]^ More importantly, most scintillators surfer serious afterglow problems, which often causes an ultra‐long radiative lifetime and device response time.^[^ ^24^, ^25^ ^]^ Foreign ions doping partially solved this problem, but limited ions matched the energy level and crystal lattice of host scintillators.^[^ ^26^, ^27^, ^28^ ^]^ Perovskite scintillators were discovered possessing high quantum yield, tunable emission peak position, and little self‐absorption with large stokes shift due to the self‐trapped excitons (STE) effect.^[^ ^29^, ^30^ ^]^ However, the STE process also causes serious afterglow phenomenon with slow charge carrier recombination velocity and long recombination lifetime of microseconds or longer. Therefore, it is still challenging for perovskite X‐ray detectors to compete with traditional X‐ray detectors in comprehensive practical performance.

In this article, we developed a novel hybrid perovskite X‐ray detector by combining direct‐type methylamine lead iodide (MAPbI_3_) semiconductor with indirect‐type zero‐dimensional cesium copper iodide (Cs_3_Cu_2_I_5_) scintillator, which can both effectively attenuate the X‐rays and generate collectible charge carriers. The mixed powders were tableted through a high pressure of 0.3 GPa, and the hybrid wafer showed a fast response time of 36.3 ns through the energy transfer process, which is nearly 30 times shorter than the scintillation afterglow of 1.07 µs for pure Cs_3_Cu_2_I_5_ scintillator. Fast X‐ray detection capability is also demonstrated by the hybrid detector arrays array in a large device area, and corresponding spatial resolution is also consistent with the pixel size, avoiding the isotropic light scattering effect of a scintillator.

## 2 Results and Discussion

### 2.1 Energy Transfer‐Assisted Fast Device Response

**Figure 1:**
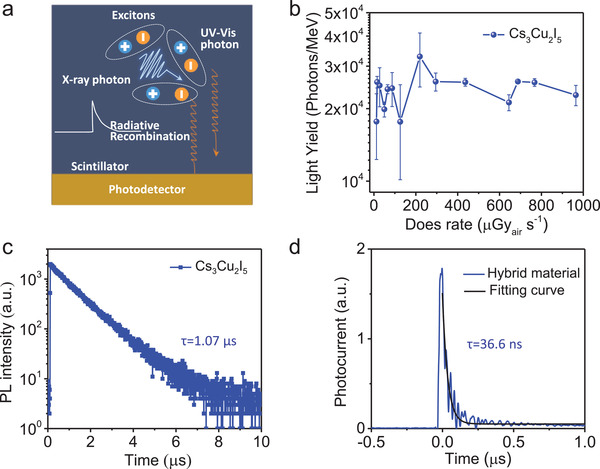
a) Schematic diagram of Cs_3_Cu_2_I_5_ scintillator light yield test. b) Light yield of Cs_3_Cu_2_I_5_ at various X‐ray dose rates under irradiation of hard X‐ray with peak voltage of 120 kV_p_. c) Time‐resolved PL decay dynamics of Cs_3_Cu_2_I_5_. d) The transient photocurrent decay curve of the hybrid material device.

Dimer polyhedrons of [Cu_2_I_8_]^6–^ were spatially isolated by the surrounding inorganic Cs^+^ ions in Cs_3_Cu_2_I_5_ crystal, and the Jahn–Teller distortion of polyhedrons in their crystal structure resulted in a strong STE effect, which brings bright blue photoluminescence (PL) with a quantum yield up to 89%. Thus Cs_3_Cu_2_I_5_ crystal can serve as scintillator material to convert the high‐energy X‐ray photons into UV–vis light (see **Figure** **1**a).^[^ ^31^ ^]^ Light yield (LY) is one of the most important figure‐of‐merit for a scintillator, and the LY of Cs_3_Cu_2_I_5_ is directly measured by assuming that the emission intensity of the scintillation process is uniform in all directions. The LY of Cs_3_Cu_2_I_5_ was derived according to the following equations:

(1) $$N_{1}=\frac{\left(I_{0}\times S_{1}\times t\right)}{h\upsilon }$$

(2) $$N_{2}=\frac{\left[\left(D\times \Phi \right)/\mu_{m}\right]\times \left(S_{1}\times t\right)}{E}$$

(3) $$LY\;=\frac{N_{1}}{N_{2}}$$

where *N_1_* is the number of photons emitted by a scintillator, *I_0_* is the emission light intensity, *hυ* is the emission photon energy, *S_1_* is the surface area of a sphere with a radius of the distance between the silicon diode photodetector and emission center, *t* is scintillation time, *N_2_* is the theoretically produced photon numbers by one X‐ray photon, *D* is X‐ray dose rate, *Φ* is the X‐ray absorption rate of the sample, *µ_m_* is mass attenuation coefficient, and *E* is the average photon energy of X‐rays. The LYs of Cs_3_Cu_2_I_5_ under different X‐ray dose rates are roughly calculated and shown in the Figure 1b, and the LY of Cs_3_Cu_2_I_5_ is independent of the exposed dose rate of X‐rays, and exhibits an average value of 26 000 photons MeV^−1^, close to the reported value of 29 000 ± 580 photons MeV^−1^ (see Supporting Information for details),^[^ ^32^ ^]^ indicating the high quality and good reproducibility of synthesized Cs_3_Cu_2_I_5_ scintillator. Scintillator decay time is another important figure‐of‐merit for a scintillator to determine the detector response time. The short decay time of tens of nanoseconds is necessary for dynamic medical CT imaging during multiple‐angle‐scanning. Figure 1c shows that the time‐resolution photoluminescence (TRPL) spectrum of Cs_3_Cu_2_I_5_ gives a very long decay lifetime of 1.07 µs, agreeing with many reported perovskite scintillators due to the STE effect,^[^ ^30^, ^31^ ^]^ which is still limiting perovskite scintillators to step forward toward commercialization.

**Figure 2:**
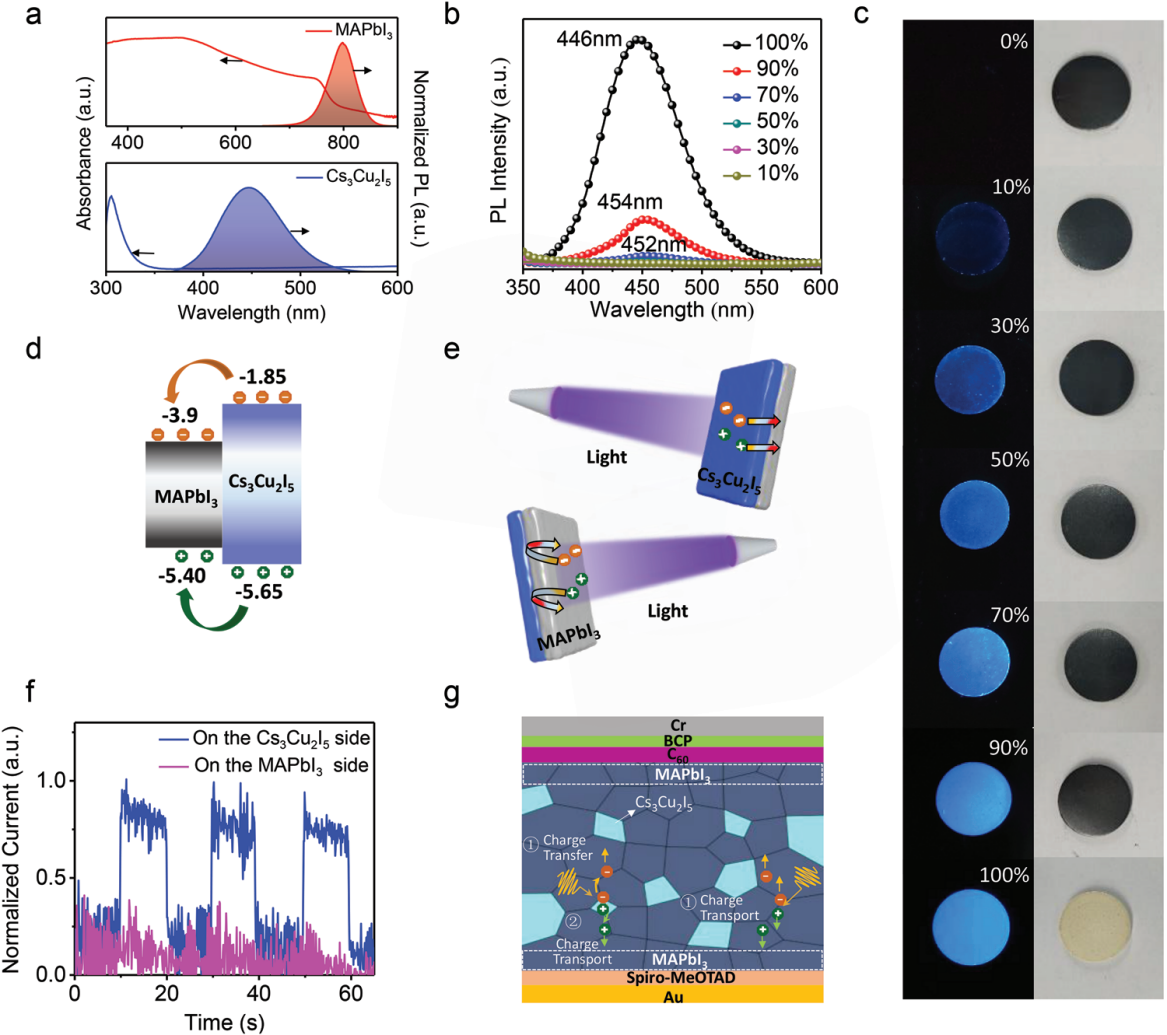
a) UV–vis absorption and PL spectrum of MAPbI_3_ (top) and Cs_3_Cu_2_I_5_ (bottom). b) PL spectra of hybrid material with different scintillator contents. c) Photographs of hybrid material with different scintillator contents under 254 nm UV excitation (left) and ambient light (right). d) Energy level structure of MAPbI_3_ and Cs_3_Cu_2_I_5_, respectively. e) Schematic design to verify the energy transfer process. f) The photocurrent on–off signal as the light is irradiated from Cs_3_Cu_2_I_5_ and MAPbI_3_ sides, respectively. g) Schematic mechanism of two types charges transport paths based on hybrid devices.

Considering Cs_3_Cu_2_I_5_ and MAPbI_3_ are both responsive to X‐rays, cheap, easily available raw materials, and simple synthesized routes, we used the tablet compression method to fabricate direct and indirect hybrid X‐ray detectors, which cannot only save the crystals growth time, but also achieve large area flat panel by simply employing large‐sized molds.^[^ ^33^ ^]^ More importantly, our hybrid X‐ray detector dramatically reduced the X‐ray response time from scintillation afterglow lifetime of 1.07 µs to 36.6 ns in the photocurrent response tests (see Figure 1d). We speculated that there is a fast energy transfer process between Cs_3_Cu_2_I_5_ and MAPbI_3_. In order to verify the energy transfer process, we first performed the absorption and PL spectra of the MAPbI_3_ and Cs_3_Cu_2_I_5_ films, respectively (see **Figure** **2**a). PL spectra of hybrid material with different scintillator contents were shown in Figure 2b, and PL intensity was quenched to 80% of the original intensity when 10% MAPbI_3_ semiconductors were added, the PL intensity of Cs_3_Cu_2_I_5_ is efficiently quenched with only a small doping ratio (Figure S2, Supporting Information), demonstrating the energy transfer from Cs_3_Cu_2_I_5_ scintillator to MAPbI_3_. Figure 2c displays the corresponding photos of tableted wafers with different Cs_3_Cu_2_I_5_ contents, which is also consistent with the PL study. We also described the energy levels of Cs_3_Cu_2_I_5_ scintillator and MAPbI_3_ semiconductor in Figure 2d,^[^ ^34^, ^35^ ^]^ and the two materials can form heterojunction and present a type I alignment in energy levels, which can promote energy transfer from the Cs_3_Cu_2_I_5_ scintillator and the MAPbI_3_ semiconductor. To further demonstrate the energy transfer process, we prepared planar Cs_3_Cu_2_I_5_ and MAPbI_3_ heterojunction by tableting two thin plates together, and top and bottom surfaces were thermal evaporated semitransparent Au electrodes. The device current was inspected without any applied bias as single Cs_3_Cu_2_I_5_ or MAPbI_3_ was excited by short wavelength light in Figure 2e to evaluate the charges self‐diffusion and transfer processes. When charges were excited on the Cs_3_Cu_2_I_5_ side, the device current showed an on‐off signal switch in Figure 2f. It should be noted that there is no bias applied on the device, indicating that charges self‐diffused from Cs_3_Cu_2_I_5_ to MAPbI_3_. While if charges are excited on the MAPbI_3_ side, there is no signal current observed due to the energy barrier of type I heterojunction. The experimental results confirmed the energy transfer process from Cs_3_Cu_2_I_5_ to MAPbI_3_. In addition, we also performed the photoluminescence excitation spectra (PLE) and PL spectrum for MAPbI_3_, Cs_3_Cu_2_I_5_, and hybrid samples (Figure S3, Supporting Information). The PLE spectra of the hybrid sample monitored at 780 nm show a prominent excitation peak of Cs_3_Cu_2_I_5_ (280–320 nm), consistent with the PLE spectrum of Cs_3_Cu_2_I_5_, indicating the energy transfer from Cs_3_Cu_2_I_5_ to MAPbI_3_. Another evidence is shown from the PL spectrum, and the fluorescence of Cs_3_Cu_2_I_5_ in the hybrid sample was quenched, while the fluorescence of MAPbI_3_ in the hybrid sample was enhanced at the same condition_._ This also indicates an energy transfer process from the Cs_3_Cu_2_I_5_ to MAPbI_3_, in consistent with the emerging fast radiative recombination path shown in PL lifetime characterization in Figure S4 (Supporting Information).

### 2.2 Device Working Mechanism of Hybrid X‐Ray Detector

**Figure 3:**
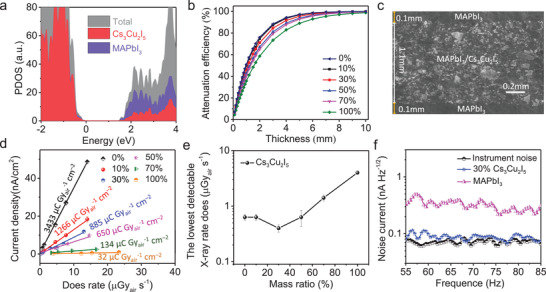
a) Projected density of states (PDOS) of hybrid material. b) Attenuation efficiency of hybrid material with different scintillator contents to 120 keV_p_ X‐ray photons (in terms of the photoelectric effect) versus thickness. c) SEM image of the hybrid wafer. d) X‐ray‐generated photocurrent at various dose rates, with the sensitivity derived by fitting the slope. e) The lowest detectable X‐ray rate does of the hybrid devices with different scintillator contents. f) Noise current for the MAPbI_3_, 30%Cs_3_Cu_2_I_5_ wafer at different frequencies.

Based on the observed energy transfer process, we proposed two different charges collection paths both existing in the hybrid X‐ray detector. The path I is that the X‐ray photons are attenuated by the MAPbI_3_ semiconductor, and the induced charges are directly collected through the continuous MAPbI_3_ phase under applied bias (see Figure 2g). The other one (Path II) is that the charges are generated by the Cs_3_Cu_2_I_5_ scintillators, which are fast transferred to the MAPbI_3_ phase before radiative recombination, followed by charges collection through the path I. The energy transfer efficiency estimated from the PL fluorescence quenching is always more than 90% if the MAPbI_3_ phase can maintain 20% in hybrid sample (Figure S5, Supporting Information), indicating that most of the excitons generated by scintillation under X‐ray are transferred to MAPbI_3_ before radiative recombination. This assumption only stands when the two phases of the Cs_3_Cu_2_I_5_ scintillator and the MAPbI_3_ semiconductor are stable with no phase transition. In order to further explore whether phase separation or ions exchange occurs between MAPbI_3_ and Cs_3_Cu_2_I_5_, we performed X‐ray powder diffraction (XRD) measurements of hybrid samples with different mixed ratios, and no unexpected diffraction peak was observed (see Figure S6, Supporting Information),^[^ ^36^ ^]^ indicating that there was no ions exchange or phase transition phenomenon between MAPbI_3_ and Cs_3_Cu_2_I_5_. In addition, energy dispersive X‐ray spectroscopy (EDX) results also confirmed that Cs^+^ and Pb^2+^ were uniformly distributed in the hybrids, and there was no phase separation (see Figure S7, Supporting Information). These results indicate that MAPbI_3_ has good stability after blending with Cs_3_Cu_2_I_5_, which provides a good opportunity for X‐ray detection. First‐principle density functional theory (DFT) calculation was also carried out to analyze the energy band structure of the hybrid materials. The calculations indicate that the hybrid material possesses direct bandgaps of 1.6 eV at the Γ point, which is in good consistent with the reported bandgaps (1.5 eV) of MAPbI_3_.^[^ ^37^ ^]^ It can also be clearly seen that the introduction of Cs_3_Cu_2_I_5_ does not introduce additional trap states within the bandgap of MAPbI_3_ (see **Figure** **3**a).

Considering the escaped PL emission from hybrid tablets surface, we covered a thin layer of MAPbI_3_ on both sides of the Cs_3_Cu_2_I_5_–MAPbI_3_ hybrid layer, so that the charges generated by Cs_3_Cu_2_I_5_ can be completely transferred to the MAPbI_3_ phase. In order to fairly compare the X‐ray detection performance, we first examined the attenuation efficiencies of the hybrid materials with different mixed ratios to ensure the same attenuation efficiencies with comparable charge carriers generated by finely tuning the thickness of hybrid layers in Figure 3b. Since hard X‐rays (tube voltage, 80–120 kV_p_) are more frequently used for medical imaging, we focused on the device response to hard X‐rays with an accelerating voltage of 120 kV_p_. Materials attenuation equation can be described as follows:^[^ ^38^ ^]^

(4) $$I=I_{0}\times e^{-\mu_{m}\rho l}$$

where *I* is the transmitted photon flux intensity, *I_0_* is the incident photon flux intensity, *µ_m_* is the mass attenuation coefficient, *ρ* is the density of the material, and *l* is the photon interaction length with materials. The required thickness of hybrid layers gradually increases a little bit as the increase of the Cs_3_Cu_2_I_5_ contents, since the X‐ray absorption coefficient of MAPbI_3_ is larger than that of Cs_3_Cu_2_I_5_.

### 2.3 X‐Ray Detection Performance of Hybrid Device

One typical scanning electron microscopy (SEM) image of the hybrid wafer is shown in Figure 3c, and the MAPbI_3_–Cs_3_Cu_2_I_5_ mixture was sandwiched between MAPbI_3_ layers. The SEM image clearly shows the layered structure without any interpenetration. Through EDX measurement, it can be seen that there is no phase transform after mixing these two materials together and the Cs_3_Cu_2_I_5_ grains are uniformly distributed among the MAPbI_3_ grains boundaries. (Figure S8, Supporting Information). We then constructed the device structure of Au/Spiro‐MeOTAD/MAPbI_3_/MAPbI_3_–Cs_3_Cu_2_I_5_/MAPbI_3_/C_60_/bathocuproine (BCP)/Cr. The lowest detectable dose is one of the most important figures‐of‐merit for X‐ray detection during medical CT imaging, since exposed high dosage may cause disease like cancer. Although a high sensitivity of MAPbI_3_ wafer is often observed as shown in Figure 3d, which is beneficial for a lower detection limit, the device noise level is paramount of importance to reduce the detection limit. The sensitivity of the hybrid devices gradually decreases as the increase of Cs_3_Cu_2_I_5_ contents. The 30% Cs_3_Cu_2_I_5_ device has the lowest detection dose rate of 0.41 μGy_air_ s^–1^, much lower than the practical dose rate of 5.5 μGy_air_ s^–1^ during CT imaging.^[^ ^39^ ^]^ This value is also 1.5 times lower than the direct‐type MAPbI_3_ semiconductor, and 10 times lower than that of the Cs_3_Cu_2_I_5_ scintillator as summarized in Figure 3e. Considering the electrical signals are all output through the MAPbI_3_ phase, and the charges recombination velocity should be the same, thus the electrical signals contribution can be simply determined by the stopping power difference of two phases and energy transfer efficiency between them (see Figure S9, Supporting Information). In addition, to confirm the noise level difference between the 30% Cs_3_Cu_2_I_5_ device and MAPbI_3_ device, we measured the noise current by Fast Fourier Transformation (FFT) analyzer coupled with a low noise current amplifier, and the noise current of 30% Cs_3_Cu_2_I_5_ device is obviously lower than the direct‐type MAPbI_3_ device, and close to the instrument noise (see Figure 3f).

**Figure 4:**
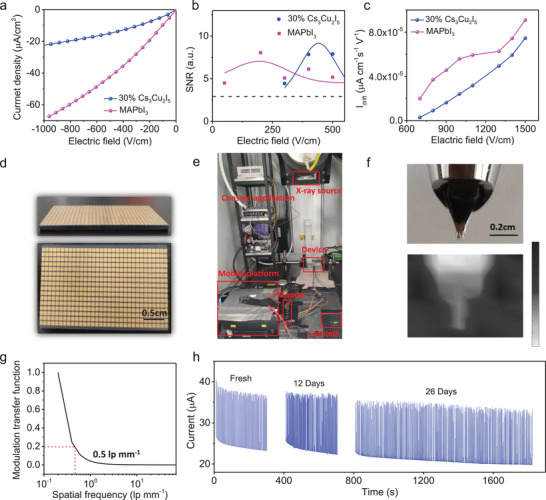
a) The resistivity of 30% Cs_3_Cu_2_I_5_, MAPbI_3_ wafer, respectively. b) SNR of 30% Cs_3_Cu_2_I_5_ and MAPbI_3_ devices, respectively. c) Ion migration for the 30% Cs_3_Cu_2_I_5_, MAPbI_3_ device under different electric field intensities, respectively. d) Optical image of the hybrid wafer array. e) Home‐made X‐ray imaging setup. f) Optical image (up) and the corresponding X‐ray image (down) of a pen nib. g) Modulation transfer function for the fabricated detector array. h) Long‐term operation stability tests of 30% Cs_3_Cu_2_I_5_ detector in response to X‐ray pulses, and the devices are stored for 0 days, 12 days, and 26 days before testing.

The suppressed noise current should result from the increased bulk resistivity, and **Figure** **4**a shows that the bulk resistivity of 30% Cs_3_Cu_2_I_5_ wafer is 5.4 × 10^7^ Ω cm, ≈1.21 times larger than that of the MAPbI_3_ wafer. It should be noted that although the bulk resistivity of the Cs_3_Cu_2_I_5_ wafer is over two orders magnitude larger than the MAPbI_3_ wafer, signal and device noise can both influence the device X‐ray detection limit. We directly characterized the device SNR to see the difference between MAPbI_3_ wafer and hybrid wafer. It can be clearly seen that the hybrid wafer has improved SNR compared with the MAPbI_3_ wafer at high electric field range (see Figure 4b), and this should result from the suppressed dark current drift and low device noise of the hybrid material. The dark current drift in perovskite material under bias is closely related to the ions migration phenomenon, which is one of the figures‐of‐merit for an ionizing radiation detector because ions migration will lead to a baseline drift, and reduce the SNR and the lowest detectable dose rate. To quantitatively characterize the ionic migration, we recorded the dark current drift versus time at the operation electric field. As shown in Figure 4c, ions migration of MAPbI_3_ device is more serious than that of 30% Cs_3_Cu_2_I_5_ device under same electric field. The suppressed ions migration can be attributed to that the ions mobile paths were blocked by the Cs_3_Cu_2_I_5_, which existed at the grain boundaries of the MAPbI_3_.

Fast X‐ray detection is one of the major advantages of the hybrid X‐ray detector. The fast response speed can save the X‐ray imaging time, prolong the service lifetime of X‐ray imaging system, and enable the dynamic imaging capability. We fabricated a 3.6 cm × 2.4 cm large tablet hybrid detector array with 512 pixels on it as shown in Figure 4d. All the pixels were preconnected to line up the anode poles, and the cathode was shared by totally covered electrode with a conduction wire connected (see Figure 4e). A pen nib was employed as a target subject for fast imaging in Figure 4f, and an X‐ray image can be obtained within seconds by moving our mobile platform to read each linear signal of the array. Since the response time of the hybrid device is 36.6 ns, and the total data collection time of the imaging array is ≈18.7 µs, which highlights the advantages of fast X‐ray imaging capability. The response time can be further shortened if the detector arrays are integrated with the silicon FET or CMOS circuits to read all the pixels signals at the same time. In addition, we also evaluated the spatial resolution of the hybrid device arrays. The well‐acknowledged slanted‐edge method was adopted for the modulation transfer function (MTF) measurement. The response to the edge was recorded as the edge spread function (ESF), which was differentiated to obtain the line spread function (LSF), as shown in Figure S10 (Supporting Information). Then, the MTF could be derived by applying a fast Fourier transformation to the LSF.^[^ ^40^ ^]^ The resulting MTF is presented in Figure 4g, and the spatial resolution is ≈0.5 lp mm^–1^ at 20% MTF value, consistent with the pixel size, which is widely employed for fast medical CT imaging. In addition, we compare the stability of the electrical signal output by the hybrid device and the MAPbI_3_ device under continuous X‐ray exposure for 2 h as shown in Figure S11 (Supporting Information). The results show that the hybrid device exhibits a stable response to hard X‐ray pulses with no SNR loss, indicating that the operational stability is improved compared with the MAPbI_3_ device at ambient conditions without any encapsulation. Moreover, the long‐term operation stability tests of the hybrid devices in response to X‐ray pulses are also performed, and the devices without any encapsulation are stored for 0, 12, and 26 days before testing, and the hybrid device also exhibited excellent operational stability. (see Figure 4h).

## 3 Conclusion

In summary, we developed a novel direct–indirect hybrid perovskite wafer through a fast‐tableting process for X‐ray detection. Due to the fast energy transfer from the Cs_3_Cu_2_I_5_ scintillator to the MAPbI_3_ semiconductor, the response time of hybrid devices can be sharply shortened for overcoming the intrinsic long scintillation afterglow. We also demonstrated the fast X‐ray detection capability within seconds, which laid the foundation for rapid imaging. What's more, compared with an indirect‐type scintillator, the hybrid detector cannot only avoid the energy loss caused by the intermediate energy conversion process, but also solve the isotropic light scattering effect of a scintillator, making the large area panel more convenient, low cost, and compatible with commercial production. The sensitivity of the hybrid X‐ray detector is improved by 28 times compared with the indirect‐type scintillator, and the lowest detectable dose rate is also reduced by 10 times. Compared to the direct‐type semiconductor, the ions migration effect of MAPbI_3_ semiconductor is obviously suppressed, which results in an improved SNR and a lower detectable dose rate of the hybrid X‐ray detector. The spatial resolution of 0.5 lp mm^–1^ matches the pixel size of the detector array, avoiding the light scattering and reabsorption issues of a scintillator. In addition, the hybrid X‐ray detector also exhibits excellent operation stability under a working electrical field with no SNR loss after about 1 month of storage, showing strong competition in large area medical CT imaging applications in the future. To further improve the device performance, we can focus on energy transfer increment by elevating device working temperature; reasonably designing the materials compositions and band structure; tailoring the phase distribution of mixed scintillators and direct semiconductor wafers. The hybrid perovskite X‐ray detector combines the advantages of direct‐type semiconductor and indirect‐type scintillator, and serves as a good complementary to the existing X‐ray detectors.

## 4 Experimental Section

### Materials

Cuprous iodide (CuI, 99.95% metals basis), Lead iodide (PbI_2_, 99.9%), and *γ*‐Butyrolactone (GBL, ≥99%) were purchased from Aladdin. Cesium iodide (CsI, 99.9%) was purchased from Energy Chemical, and *N*,*N*‐dimethylformamide (DMF, ≥99.0%) was purchased from Sinopharm. All these chemicals were used without any further purification.

### Growth of MAPbI_3_ and Cs_3_Cu_2_I_5_ Single Crystals

The synthesis of methylamiodine (MAI) was reported in the literature.^[^ ^41^ ^]^ In order to grow MAPbI_3_ single crystal, 4.292 g PbI_2_ and 12.447 g MAI were dissolved in 30 mL GBL with the molar ratio of 1:1. Then the solution was stirred under 80 °C to make a clear solution, after which the solution was filtered with 0.22 µm filter immediately. Then the solution was heated up to 120 °C, and After 1 h, one irregular crystal formed on the bottom of the solution slowly. Finally, the large MAPbI_3_ single crystal was obtained. The 0D Cs_3_Cu_2_I_5_ crystals were prepared at room temperature by the solvent evaporation crystallization process. In order to grow Cs_3_Cu_2_I_5_ single crystal, 0.572 g CuI and 1.169 g CsI were dissolved in 6 mL DMF with the molar ratio of 2:3, then placed in a Petri dish, evaporate, and dry naturally, and then clean it with DMF to get Cs_3_Cu_2_I_5_ single crystal.

### Wafer Fabrication

MAPbI_3_ and Cs_3_Cu_2_I_5_ crystals were ground with a mortar and pestle for 30 min to achieve uniform mixed powders. First, the mixed powders were mounted into a pie shape mold through a compressor, and subsequently were subjected to a pressure of 0.3 GPa through a hydraulic press (FW‐4A) for 5 min. The targeting thickness could be obtained by adjusting the precursor loading amount. The wafer was then annealed on the hot plate at 100 °C for 10 min in the air for further crystallization. The area of the wafer is 1.326 cm^2^.

### Photodetectors Wafer Device Fabrication

The electron transport layers C_60_ (30 nm) and BCP (10 nm) were sequentially deposited on the surface of the active layer by thermal evaporation, followed by depositing 50 nm Cr cathode under 5 × 10^−4^ Pa. Then Spin coating a layer of Spiro‐MeOTAD (30 mg mL^−1^, chlorobenzene) and thermal deposition of 50 nm Au anode on the bottom surface of the corresponding wafer, respectively.

### Characterization

The XRD measurement was performed by Rigaku X‐ray diffractometer (SmartLab 3). The UV–vis spectra of the wafers were collected by UV‐2600 spectrophotometer operating from 200 to 800 nm. The steady‐state PL of Cs_3_Cu_2_I_5_ doping with different mass ratios was measured with RF‐6000 with excitation light of 320 nm. The time‐resolved PL of the hybrid samples was measured by FLS980 with a 280 nm excitation source. The PL decay signals of hybrid samples were monitored at the emission maxima of Cs_3_Cu_2_I_5_, and a bandpass filter was applied before the detector to ensure that the fluorescence generated was from Cs_3_Cu_2_I_5_. The noise current of the devices was measured under dark at the different frequencies at −100 V reverse bias and the data were recorded by the SR830 DSP lock‐in amplifier. The X‐ray detection was carried out under X‐ray irradiation with an energy of 120 keV_p_. The acceleration voltage of the X‐ray tube was 120 kV, and the peak photon energy was thus 120 keV_p_. The operating current of the X‐ray tube was tuned to 50 mA. The radiation dose rate was calibrated with a commercial dosimeter from Germany (IBA, MagicMax Universal, XR Multidetector). To test the stable operation conditions, the X‐ray detector based on the hybrid device was stored in ambient air condition at room temperature with the humidity of ≈30%, and no encapsulation was applied. In X‐ray sensitivity testing of devices, error bars were used to represent the distribution of data, where the height of the error bars was ±standard error. Three groups of the same conditions were taken to get the current signal value, then its average value was taken to calculate its standard error. All statistical analyses were performed in Origin 9.0.

## Conflict of Interest

The authors declare no conflict of interest.

## Author Contributions

H.W. conceived and supervised the project. L.L. grew single crystals, fabricated the detectors, measured the optoelectronic properties, and X‐ray detectors sensitivity. W.L. contributed to optoelectronic measurements and theoretical DFT calculation. X.F. assisted in drawing the mechanism diagram. All authors analyzed the data. H.W. and L.L. wrote the manuscript, and all the authors commented and reviewed the manuscript.

## Supporting information

Supporting Information

## Data Availability

The data that support the findings of this study are available from the corresponding author upon reasonable request.
