# Supplementary material for: Energy Transfer Assisted Fast X‐ray Detection in Direct/Indirect Hybrid Perovskite Wafer
Source: Adv Sci (Weinh). 2022 Mar 23;9(15):2103735. doi: 10.1002/advs.202103735 (PMC9130882; doi:10.1002/advs.202103735)
Supplement: Supplementary file 1 — Supporting Information [file ADVS-9-2103735-s001.pdf]

ADVANCED SCIENCE
Open Access

Supporting Information

for *Adv. Sci.*, DOI 10.1002/advs.202103735

Energy Transfer Assisted Fast X-ray Detection in Direct/Indirect Hybrid Perovskite Wafer

Lulu Liu, Weijun Li, Xiaopeng Feng, Chunjie Guo, Huimao Zhang, Haotong Wei and Bai Yang*

Supporting Information

Energy Transfer Assisted Fast X-ray Detection in Direct/Indirect Hybrid Perovskite Wafer

Lulu Liu, Weijun Li, Xiaopeng Feng, Chunjie Guo, Huimao Zhang, Haotong Wei, and Bai Yang*

X-ray scintillation efficiency

To quantify the X-ray scintillation property, we placed the pressed $Cs_3Cu_2I_5$ wafer on the silicon diode, then adjusted the X-ray dose rate by changing the thickness of different lead plates, and corrected the X-ray dose rate with a commercial X-ray dosimeter. When the X-ray dose rate is applied, the X-ray will penetrate the scintillator surface and generate excitons inside. Then the $Cs_3Cu_2I_5$ scintillator emits blue fluorescence and is collected by the silicon diode. Finally, the pulse current signal is output through the external circuit. The photocurrent of the $Cs_3Cu_2I_5$ scintillator at different X-ray dose rates is converted into the corresponding light intensity. In detail, according to the following equations:

$$I_0 = \frac{I_s}{R \times S} \tag{S1}$$

$$R = \frac{q \times EQE_{si}}{hv} \tag{S2}$$

where I_0 is the light intensity, I_s is the photocurrent of a scintillator, S is the area of the silicon diode photodetector, R is the responsivity, q is the quantity of electric charge. EQE_{Si} is the quantum efficiency of the silicon diode photodetector, h is the Planck

constant, v is the frequency of light. The photocurrent obtained by the $Cs_3Cu_2I_5$ scintillator at different X-ray dose rates is converted into the corresponding light intensity. Then, we take the different intensities of light of the scintillator through equation (1), equation (2), equation (3) to the light yield of the scintillator.

DFT calculation

The first-principles calculations were carried out via the Vienna Ab initio Simulation Package. The electron-ion interaction was described using projected augmented-wave pseudopotentials. The generalized gradient approximation formulated by Perdew-Burke-Ernzerhof was performed as the exchange-correlation function. The atomic positions were fully relaxed until the force on each atom was smaller than 0.01 eV and the convergence threshold for the self-consistent field was 10^{-4} eV.

A surface slab was modeled to explore the energy transfer, containing a three-layer $MAPbI_3$ framework and a $Cs_3Cu_2I_5$ molecule. The optimized differential charge density of the $Cs_3Cu_2I_5$ on the $MAPbI_3$ system shows obvious electrons deficient states of $Cs_3Cu_2I_5$ after contacting with $MAPbI_3$ crystals, of which electrons clouds density increases as shown in Figure S1. This also indicates that the charge is transferred from $Cs_3Cu_2I_5$ to $MAPbI_3$.

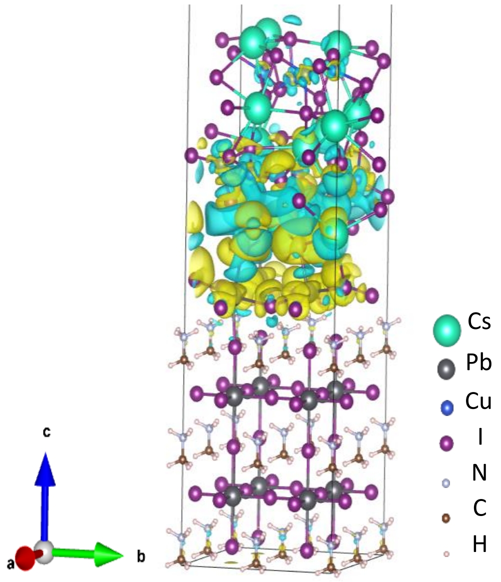

Figure S1. Differential charge density of the $Cs_3Cu_2I_5$ molecule on $MAPbI_3$.

As shown in the Figure S2, when 10% $MAPbI_3$ is doped, $Cs_3Cu_2I_5$ fluorescence is quenched by about 80%. The fast PL quenching of $Cs_3Cu_2I_5$ as the doping ratio shows the efficient energy transfer process.

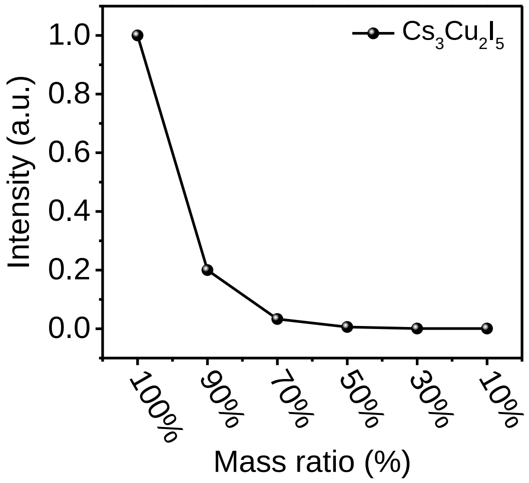

Figure S2. Normalized PL intensity of $Cs_3Cu_2I_5$ from hybrid samples with different mass ratios.

Figure S3a and S3b show the PLE spectra of $Cs_3Cu_2I_5$, $MAPbI_3$ and hybrid samples, and Figure S3c presents the PL spectra of the three samples.

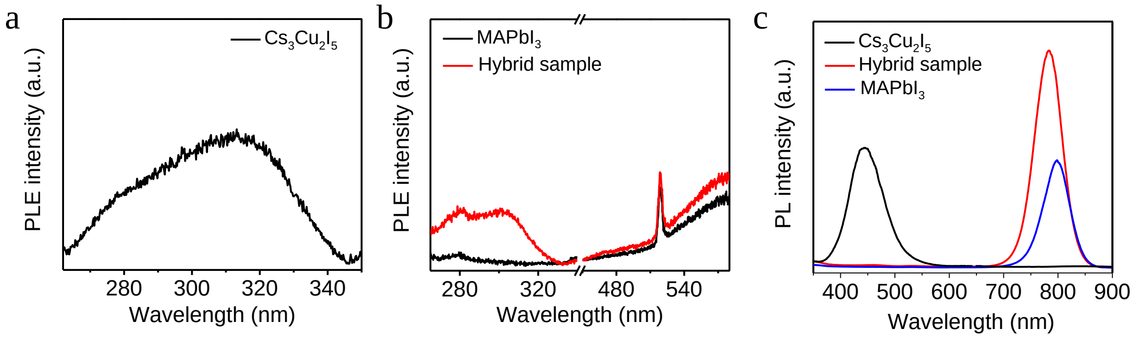

Figure S3. (a) PL excitation spectra of $Cs_3Cu_2I_5$ monitored at 450nm. (b) PL excitation spectra of $MAPbI_3$ and the hybrid sample were monitored at 780nm. (c) PL emission spectra of $MAPbI_3$, $Cs_3Cu_2I_5$ and the hybrid sample.

To demonstrate the energy transfer process from $Cs_3Cu_2I_5$ to $MAPbI_3$, we tested time-resolved PL decay dynamics of hybrid material with different scintillator contents. As the decrease of the doping ratio of $Cs_3Cu_2I_5$, two radiative recombination paths show up, and the very fast one agrees with the energy transfer process, while the other slow one has a similar PL lifetime with that of $Cs_3Cu_2I_5$. (Figure S4).

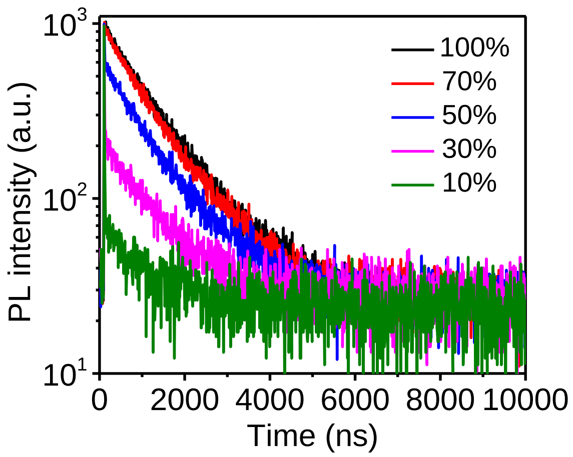

Figure S4. Time-resolved PL decay dynamics of hybrid material with different scintillator contents.

The percentage of the X-ray generated excitons in the scintillator perovskite material that transfers to the $MAPbI_3$ is assessed by the percentage of scintillator PL quenching. Doping $MAPbI_3$ into the scintillator leads to the decrease of scintillator's fluorescence intensity with excitons transfer before radiative recombination.

Therefore, the percentage of excitons generated by scintillators transferred to $MAPbI_3$ is the ratio of the reduced fluorescence intensity to the theoretical fluorescence intensity of the scintillator at this ratio. The results show that the percentage of excitons transferred to $MAPbI_3$ by scintillators is more than 90% if the content of the scintillator is less than 80% (Figure S5), indicating that most of the excitons generated by scintillation under X-ray are transferred to $MAPbI_3$ before radiative recombination.

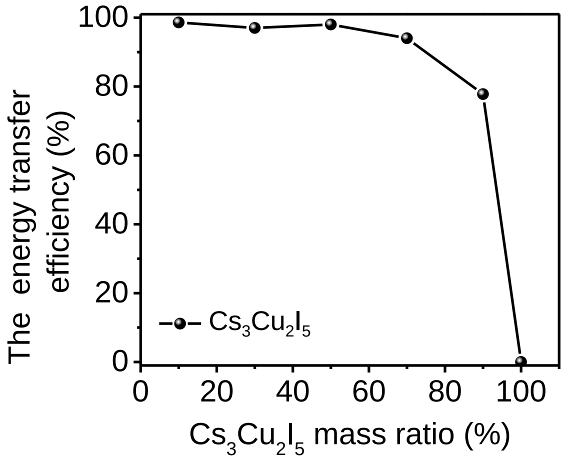

Figure S5. The energy transfer efficiency of the X-ray generated excitons in the different scintillator contents that transfer to the $MAPbI_3$.

X-ray powder diffraction (XRD) spectra of hybrid samples with different scintillator contents are shown in the Figure S6. By comparing the diffraction peaks of pure $Cs_3Cu_2I_5$ and $MAPbI_3$, no new diffraction peaks appear in the hybrid samples, indicating that there was no ions exchange or phase transition phenomenon between $MAPbI_3$ and $Cs_3Cu_2I_5$.

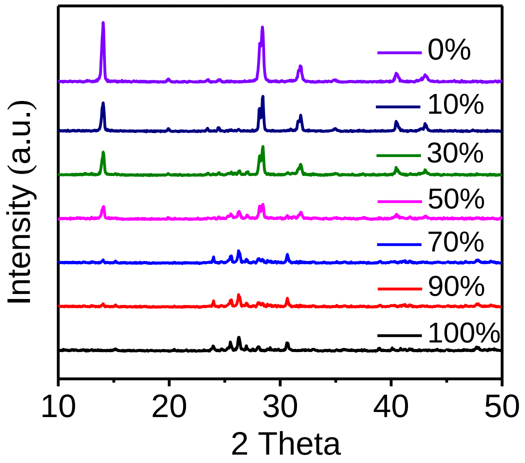

Figure S6. XRD spectra of hybrid material with different scintillator contents.

Figure S7 displays the cross-sectional energy-dispersive X-ray spectroscopy (EDX) mapping of the hybrid wafer. The results indicated the homogeneous distributions of Cs^+ and Pb^{2+} ions in the hybrid wafer, and there was no phase separation.

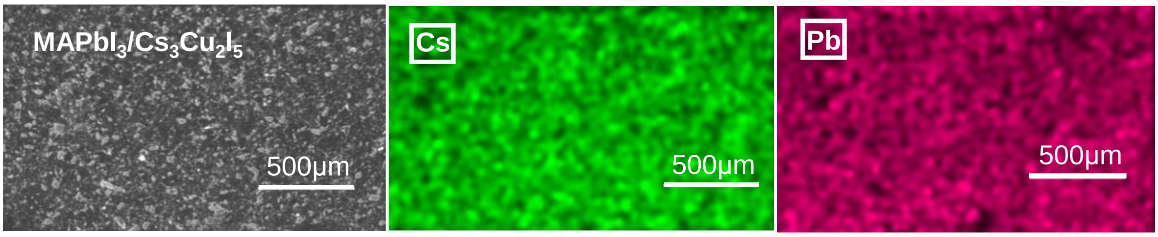

Figure S7. SEM image and EDX elemental mapping of the hybrid wafer.

Through EDX measurement, it can be seen that the $Cs_3Cu_2I_5$ grains are uniformly distributed among the $MAPbI_3$ grains boundaries as shown in Figure S8.

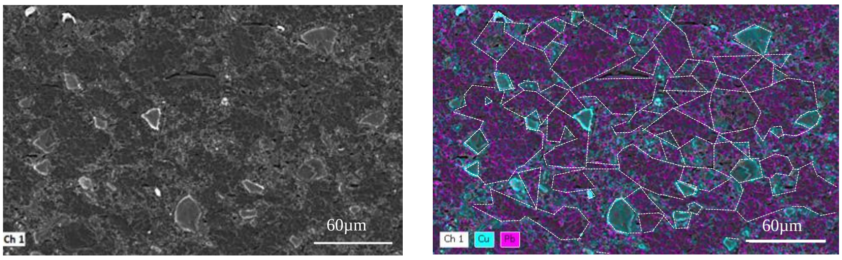

Figure S8. EDX elemental mapping of the hybrid wafer.

Since the detected electrical signal is totally collected through the $MAPbI_3$ phase, the signal contribution is only determined by their linear attenuation and energy transfer efficiency between them. The attenuation coefficient of the hybrid sample (μ') to 120 keV X-rays is calculated according to the following equation:

$$\mu' = f_C \times \mu_C' + f_M \times \mu_M' \quad \text{(S3)}$$

$$\omega_M = \frac{\mu_M' \times f_M}{\mu'} \quad \text{(S4)}$$

Where f_c is the mass fraction of $Cs_3Cu_2I_5$, f_M is the mass fraction of $MAPbI_3$, and μ_c' is the linear attenuation coefficient of $Cs_3Cu_2I_5$, μ_M' is the linear attenuation coefficient of $MAPbI_3$. ω_M is the percentage contribution of the $MAPbI_3$ to the detected electrical signals. The result of the calculation is shown in the Figure S9, as the content of $Cs_3Cu_2I_5$ increases, the contribution of $MAPbI_3$ to the detected signal gradually decreases.

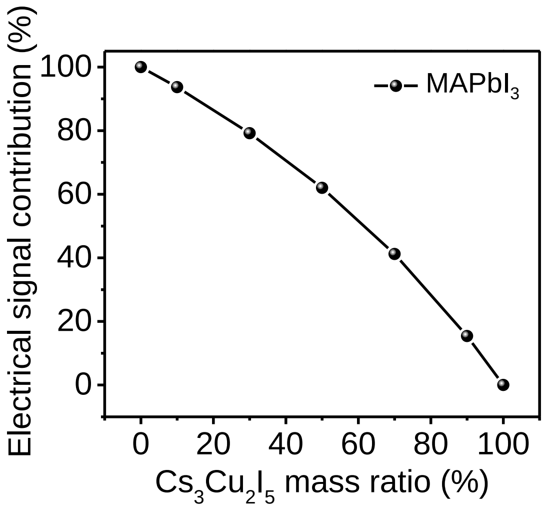

Figure S9. The percentage of $MAPbI_3$ contribution to the detected electrical signal.

We used the edge-slanted method to measure the modulation transfer function MTF. A wafer device with 1 mm ×1 mm pixel size was fixed on one direction

scanning stage and scanned a line pair edge of resolution phantom. A Keithley 2400 was used to power the device and record the photocurrent of X-ray response. Several points were fitted by a 3 order polynomial function and got edge spread function ESF(x). Then, differential function fitting and Gaussian function fitting were performed for ESF(x) to obtain LSF(x), and the fitting results are shown in Figure S10. Finally, the MTF(x) could be derived by applying a fast Fourier transformation to the LSF(x).

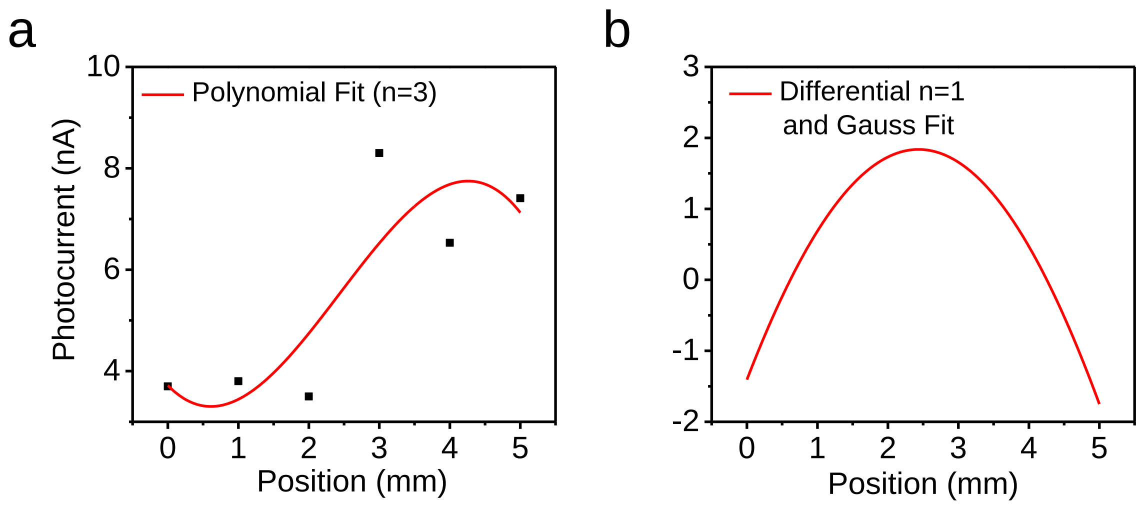

Figure S10. (a) Polynomial function fit for edge spread function (ESF(x)). (b) The derived line spread function (LSF(x)).

We compare the stability of the electrical signal output by the hybrid device and the $MAPbI_3$ device under continuous X-ray exposure for 2h, as shown in Figure S11. The results show that the hybrid device exhibits a stable response to hard X-ray pulses with no signal/noise ratio loss, indicating that the operation stability is improved compared to the $MAPbI_3$ device at ambient conditions without any encapsulation.

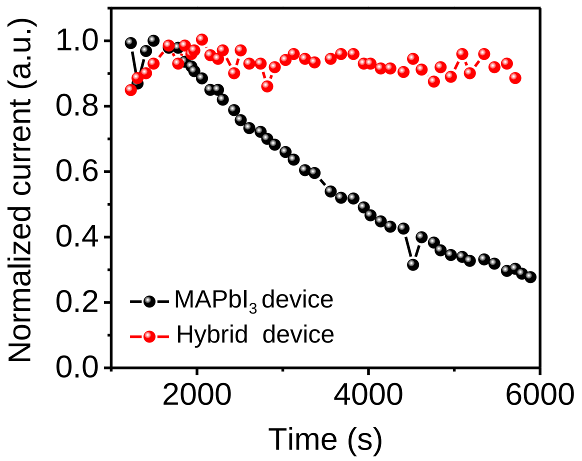

Figure S11. Operation stability tests of $MAPbI_3$ and hybrid device in response to X-ray pulses.
